# Afatinib overcoming resistance to icotinib and osimertinib in NSCLC with leptomeningeal metastasis in patients with acquired EGFR L858R/T790M or L858R/S768I mutations: Two case reports

**DOI:** 10.1016/j.heliyon.2023.e20690

**Published:** 2023-10-08

**Authors:** Guangrui Li, Mei Fang, Yazhu Zhou, Xiaocui Liu, Panpan Tian, Fengjun Mei

**Affiliations:** a Department of Infectious Diseases, The Third Hospital of Hebei Medical University, Shijiazhuang, Hebei, China; b Department of Neurology, The First Hospital of Hebei Medical University, Shijiazhuang, Hebei, China; c Department of Reproductive Medicine, The Fourth Hospital of Hebei Medical University, Shijiazhuang, Hebei, China; d Department of Neurology, The Fourth Hospital of Hebei Medical University, Shijiazhuang, Hebei, China; e Department of Neurology, North China University of Science and Technology Affiliated Hospital, Tangshan, Hebei, China

**Keywords:** Non-small cell lung cancer, Leptomeningeal metastases, Epidermal growth factor receptor, Afatinib, Case report

## Abstract

**Background:**

Advanced non-small cell lung cancer (NSCLC) is often complicated by leptomeningeal metastases (LMs), especially in patients carrying EGFR mutations. EGFR tyrosine kinase inhibitors (TKIs) are the first-line drug for patients with specific gene mutations, such as EGFR exon 19 deletion or exon 21 L858R mutation. However, after long-term TKI use, patients eventually develop drug resistance and acquire new mutations. Acquiring the EGFR T790 M mutation during TKI treatment is a marker for first/second generation TKI resistance. Osimertinib (a third-generation TKI) could overcome this resistance, especially for patients who have already developed NSCLC-LM. Treating NSCLC patients with osimertinib resistance is challenging. Our aim was to investigate whether afatinib is effective in NSCLC-LM patient who showed resistance to osimertinib. Herein, we report two patients with resistance to first- and third-generation TKIs who benefited from second-generation TKI.

**Case summary:**

Case one: A 43-year-old man was diagnosed with stage 3A NSCLC with EGFR exon 19 deletion. He underwent surgery and received 4 rounds of chemotherapy (oxaliplatin combined with liposomal paclitaxel), after which the disease was controlled by icotinib (a first-generation TKI) for 4 years. Then, he showed poor drug response and bone metastasis. A liquid biopsy was carried out, and the EGFR L858R/T790 M compound mutation was found. The patient was given osimertinib (a third-generation TKI). The patient was in a stable condition for 2 years and then he developed bilateral peripheral facial palsy. Brain MRI showed enhancement in the left temporal lobe and meninges, and cerebrospinal fluid (CSF) cytology detected tumour cells in the CSF. NSCLC-LM was diagnosed. His performance status (PS) score was 3–4. Liquid biopsy and next-generation sequencing were performed again. Different gene mutations and copy number alterations were found this time, including EGFR L858R, but without the EGFR T790 M mutation. His disease was controlled with intrathecal methotrexate and oral afatinib (a second generation TKI). The patient has shown clinical remission (PS score: 1–2) until now, which is longer than 10 months.

**Case two:**

A 50-year-old man was diagnosed with NSCLC in May 2020. He underwent one round of chemotherapy before thoracoscopic partial lobectomy of the right upper lung. Histological study of the lung tissue showed lung adenocarcinoma with the EGFR L858R mutation. Then, the disease was controlled with icotinib. One year later, he was diagnosed with NSCLC-LM. Liquid biopsy and sequencing showed an EGFR L858R/S768I compound mutation. The patient was treated with osimertinib. His condition was stable for 5 months before his central nervous system (CNS) symptoms were exacerbated. Liquid biopsy and sequencing were carried out again, and his gene mutation profile had not changed much. Then, the patient was given afatinib, and his condition has remained stable for 11 months.

**Conclusion:**

Afatinib might be suitable for NSCLC-LM patients with EGFR compound mutations who show resistance to icotinib and osimertinib, since it might help overcome first- and third-generation TKI resistance.

## 1 Introduction

Lung cancer is one of the leading causes of cancer, and the predominant type of lung cancer is non-small cell lung cancer (NSCLC) [1]. Patients with NSCLC often carry gene mutations in epidermal growth factor receptor (EGFR), especially Asian people [2,3]. In addition, NSCLC patients with EGFR mutations have a greater chance of developing leptomeningeal metastases (LMs) [4], which makes treatment challenging. NSCLC patients with EGFR activating mutations (i.e., 19del and L858R) have been successfully treated with EGFR tyrosine kinase inhibitors (TKIs), and first-generation TKIs (including icotinib, erlotinib and gefitinib) are the standard first-line therapy. However, after long-term TKI use, patients eventually acquire TKI resistance and new gene mutations. In particular, acquiring the EGFR T790 M mutation is a marker for resistance to first- and second-generation TKIs, but osimertinib, a third-generation TKI, has been successfully used to treat NSCLC-LM patients, which could overcome TKI drug resistance and has a better ability to penetrate the blood‒brain barrier (BBB) [4,5].

Diagnosis and monitoring of LM are challenging, especially for patients without radiographic and cerebrospinal fluid (CSF) cytologic findings. Liquid biopsy and circulating tumour DNA (ctDNA) sequencing is a noninvasive approach to diagnosis, and it provides valuable information for cancer management, such as detecting cancer driver mutations (e.g., TP53, EGFR, KRAS, and ROS1).

When the patient shows resistance to third-generation TKIs, chemotherapy and palliative treatment are considered suitable. It needs to be emphasized that patients with advanced NSCLC are often in poor physical condition and cannot tolerate standard chemotherapy. According to the National Comprehensive Cancer Network (NCCN) Clinical Practice Guidelines in Oncology, patients with PS > 2 are not suitable for chemotherapy or surgery, and only palliative care is considered for these patients [6].

In this study, blood sample and CSF sample were collected from the patients. Then ctDNA extraction and library construction was performed with the NucleoSpin Plasma XS kit and the KAPA Hyper DNA Library Prep Kit. Deep Sequencing is performed on Illumina HiSeq 4000 using PE75 V1 Kit. Cluster generation and sequencing is performed according to manufacturer's protocol. In total, 425 cancer related genes were listed in Supplementary Table 1.

This study presents two cases of advanced NSCLC with LM, one bearing the EGFR L858R/T790 M compound mutation and the other the L858R/S768I compound mutation, which were successfully treated by using afatinib, a second-generation TKI.

### 1.1 Case presentation

Case 1 In 2014, a 43-year-old man was diagnosed with stage IIIA NSCLC that harboured an activating EGFR exon 19 deletion mutation (based on an immunohistochemical study). He had no overt symptoms associated with the chest lesion. He had a smoking history of 7.5 years and no other notable medical history. After lung surgery was performed, he received four rounds of chemotherapy with oxaliplatin and paclitaxel liposomes. After chemotherapy, he was treated with icotinib for 4 years. In 2018, he showed bone metastasis in T4 and T5. Then, he underwent liquid biopsy and second-generation sequencing. EGFR L858R/T790 M compound mutations were found, which indicated first-generation TKI resistance. The patient was given osimertinib for 3 years.

In December 2021, he exhibited gradual limb weakness, headache, and bilateral peripheral facial palsy and developed signs of meningeal irritation. He was transferred from the oncology department to the neurology department. His PS score was 3–4. Enhanced brain MRI and lumbar puncture were carried out. Tumour cells were detected in the CSF. Enhanced lesions were detected in both the pia mater and brain parenchyma. Liquid biopsy was used again, and several gene mutations and copy number alterations were found this time, including EGFR L858R, TP53 T125Sfs*45, MRM8 S622I, PIK3C3 A719T, RB1 L700 W mutation, and CDH1/EGFR/IL-7R/RB1/RICTOR/TERT copy number variance (Table 2). However, interestingly, the EGFR T790 M mutation was not detected this time. The patient's PS score was 4, and he could not tolerate standard chemotherapy.

We tried a second-generation TKI, afatinib, combined with methotrexate and dexamethasone intrathecal injection. During the 4-week induction stage, 10 mg methotrexate and 5 mg dexamethasone were used twice a week. In the 4-week consolidation stage, intrathecal chemotherapy was organized into three phases: 4 weeks of induction (10 mg methotrexate and 5 mg dexamethasone twice a week), 4 weeks of consolidation (10 mg methotrexate and 5 mg dexamethasone once a week) and maintenance (10 mg methotrexate and 5 mg dexamethasone once a month). One month later, his PS score was 1, and his brain MRI showed a diminished dissemination lesion along the sulcus of the cerebellum.

**Fig. 1:**
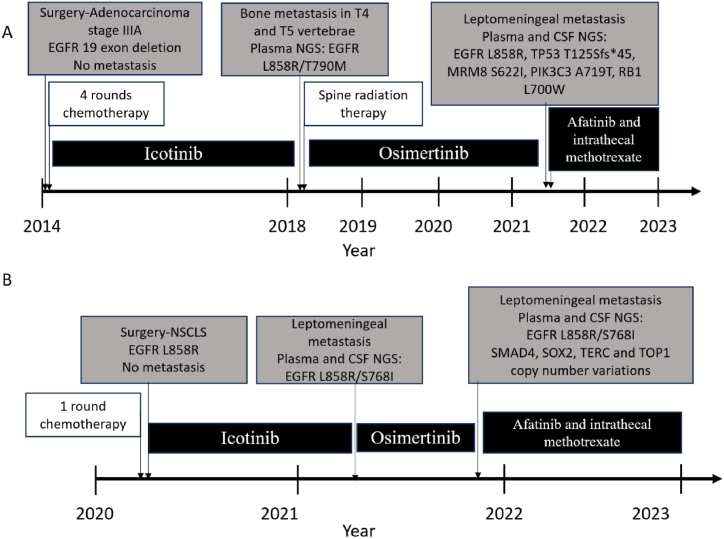
Timeline of treatments and examinations of two patients is shown.

**Fig. 2:**
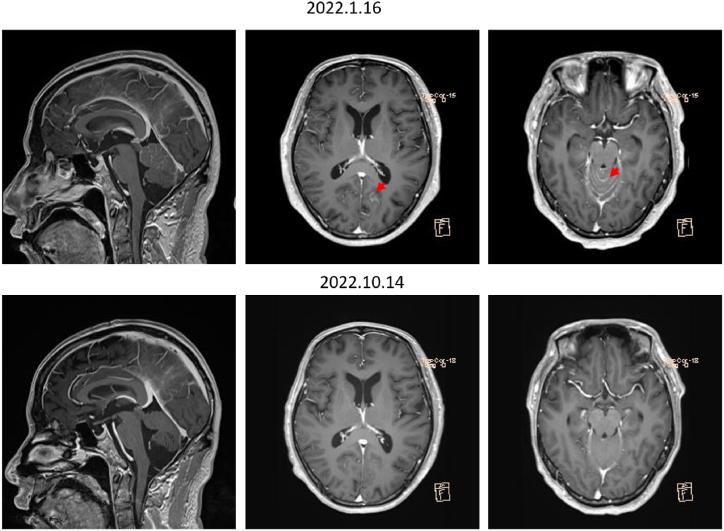
Contrast-enhanced MRI of the head with sagittal view (left panels) and axis view (middle and right panels) of patient one. The red arrow indicates meningeal dissemination. MRI, magnetic resonance imaging. (For interpretation of the references to colour in this figure legend, the reader is referred to the Web version of this article.)

The patient is still alive with good performance status at 10 months since starting the afatinib and intrathecal chemotherapy treatment. We considered that his malignant meningitis had responded well to afatinib and intrathecal chemotherapy. This treatment was continued. His PS score was 1, and he has not shown any other lesions in the brain and lungs. His main side effect from afatinib treatment is paronychia. He tolerates the current treatment well ( Fig. 1, Fig. 2, Table 1, Table 2).

**Table 1:** Characteristics of the two patients.

Characteristics	Case No. 1	Case No. 2
Gender	Male	Male
Age	53	51
Smoking status	Yes	Yes
ECOG PS at the time of LM diagnosis	3	2
Presentation of LM	5 months after NSCLC diagnosed	5 months after NSCLC diagnosed
Meningeal reinforcement	Yes	Yes
Extracranial metastasis	Bone	No
LM with brain metastases	Yes	No
Positive CSF cytology	Yes	No

**Table 2:** Gene mutation list of the two patients.

Case No. 1			Case No. 2		
First time	Second time	Third time	First time	Second time	Third time
EGFR c.2573.T > G (p.L858R)	EGFR c.2573.T > G (p.L858R)	EGFR c.2573.T > G (p.L858R)	EGFR c.2573.T > G (p.L858R)	EGFR c.2573.T > G (p.L858R)	EGFR c.2573.T > G (p.L858R)
EGFR c.2369C > T (p.T790 M)	TP53 c.374del (p.T125Sfs*45)	TP53 c.374del (p.T125Sfs*45)	EGFR c.2303G > T (p.S768I)	EGFR c.2303G > T (p.S768I)	EGFR c.2303G > T (p.S768I)
	EGFR copy number variation CN:4.9	EGFR copy number variation CN:4.2	BRIP1 c.3077C > G (p.S1026*)	BRIP1 c.3077C > G (p.S1026*)	BRIP1 c.3077C > G (p.S1026*)
	IL-7R copy number variation CN:6.3	IL-7R copy number variation CN:6.6	CDK4 copy number variation CN:9.4	CDK4 copy number variation CN:10.2	CDK4 copy number variation CN:17.9
	RB1 copy number variation CN:0.9	RB1 copy number variation CN:0.9	CHEK2 c.558_571del (p.H186Qfs*6)	CHEK2 c.558_571del (p.H186Qfs*6)	CHEK2 c.558_571del (p.H186Qfs*6)
	RICTOR copy number variation CN:6.3	RICTOR copy number variation CN:5.4	TP53 c.818G > T (p.R273 L)	TP53 c.818G > T (p.R273 L)	TP53 c.818G > T (p.R273 L)
	TERT copy number variation CN:5.7	TERT copy number variation CN:5.3	EGFR copy number variation CN:4.7	EGFR copy number variation CN:4.1	EGFR copy number variation CN:6.2
	GRM8 c.1865G > T (p.S622I)	GRM8 c.1865G > T (p.S622I)	NKX2-1 copy number variation CN:14.2	NKX2-1 copy number variation CN:11.5	NKX2-1 copy number variation CN:23.4
	PIK3C3 c.2155G > A (p.A719T)	PIK3C3 c.2155G > A (p.A719T)	RUNX1 c.624del (p.M209*)	RUNX1 c.624del (p.M209*)	RUNX1 c.624del (p.M209*)
	RB1 c.2099T > G (p.L700 W)	RB1 c.2099T > G (p.L700 W)	CDKN2A c.107C > T (p.A36 V)	CDKN2A c.107C > T (p.A36 V)	CDKN2A c.107C > T (p.A36 V)
	ETV6 c.1080G > T (p.W360C)		DICER1 c.860C > G (p.S287C)	DICER1 c.860C > G (p.S287C)	DICER1 c.860C > G (p.S287C)
	CDH1 copy number variation CN:1.0		ETV4 c.317C > A (p.P106Q)	ETV4 c.317C > A (p.P106Q)	ETV4 c.317C > A (p.P106Q)
			FOXA1 c.998A > T (p.Q333 L)	FOXA1 c.998A > T (p.Q333 L)	FOXA1 c.998A > T (p.Q333 L)
			ROS1 c.1980G > A (p.W660*)	ROS1 c.1980G > A (p.W660*)	ROS1 c.1980G > A (p.W660*)
			IFG1R c.4058G > A (p.R1353H)		SMAD4 copy number variation CN:0.8
					SOX2 copy number variation CN:3.2
					TERC copy number variation CN:3.9
					TOP1 copy number variation CN:3.8

Case 2 A 50-year-old man was diagnosed with lung cancer in May 2020. He underwent one round of chemotherapy before thoracoscopic partial lobectomy of the right upper lung. Histological study of biopsy samples showed NSCLC with EGFR L858R mutation. Then, he took icotinib (a first-generation TKI). One year later, he was diagnosed with NSCLC-LM. Liquid biopsy and second-generation sequencing showed EGFR L858R and S768I compound mutations in both blood plasma and CSF samples. The patient was switched to osimertinib.

**Fig. 3:**
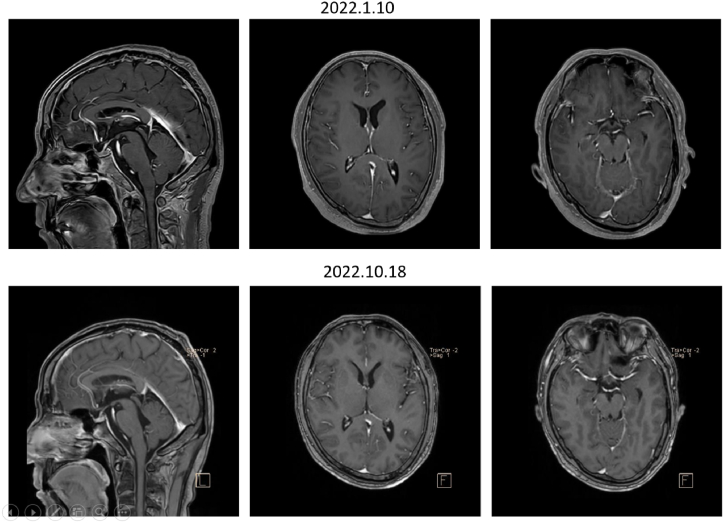
Contrast-enhanced MRI of the head with sagittal view (left panels) and axis view (middle and right panels) of patient two.

Five months later, the patient showed exacerbated CNS symptoms (headache, nausea and vomiting). Liquid biopsy and second-generation sequencing were carried out again, and his gene mutation profile had not changed much except for acquiring SMAD4, SOX2, TERC and TOP1 copy number variations. His PS score was 2, and he refused standard chemotherapy and whole brain radiotherapy. Consequently, the patient was given afatinib, and he tolerated the current treatment for 10 months and is doing well (Fig. 1, Fig. 3 , Table 1, Table 2).

## 2 Discussion

In leptomeningeal metastasis, malignant cells from the primary tumour sites were disseminated to leptomeningeal layers of the CNS. It occurs in many types of cancer, and causes a wide variety of symptoms, including headache, nausea, vomiting. Which is with dismal prognosis. Patients with lymphoma, leukemia, melanoma, non-small cell lung cancer, small cell lung cancer, and breast cancer are prone to LM [7,8]. This incidence increased in some particular groups, such as EGFR mutation positive NSCLC patients [9].

NSCLC patients with EGFR mutations are more likely to develop LM, even without CNS symptoms [10,11]. NSCLC patients with EGFR mutations should be evaluated by inquiring about their history, brain MRI and CSF cytology. However, in NSCLC-LM patients, CSF cytology and enhanced brain MRI are not always positive, which makes diagnosis and monitoring quite difficult. Liquid biopsy and ctDNA sequencing provide a sensitive method for diagnosis and can detect the tumour's gene mutation profile, including point mutations, exon deletions, exon insertions and copy number variations. ctDNA dynamics can be evaluated during patient follow-up. Monitoring ctNDA is a novel approach for the prediction of treatment response and following the disease progression [12]. In the ALEX study, the prognostic value of ctDNA was retrospectively assessed, and the researchers found that the concentration of ctDNA was positively related to the tumour size, the number of lesions, and the number of organ lesion sites [13]. There is discrepancy between tissue biopsy and liquid biopsy, which might be due to tumour heterogeneity [14]. In addition, low levels of ctDNA can lead to false negative results compared to tissue biopsies [15]. But in some situations, liquid biopsies still have advantages over tissue biopsies. For example, in one study, EGFR T790 M mutation were detected in ctDNA rather than in simultaneous tissue biopsy, but patients subsequently benefited from osimertinib therapy [16].

Many EGFR TKIs were used, the most important of which were the first-generation TKIs (icotinib, gefitinib and erlotinib), second-generation TKIs (afatinib and dacomitinib) and third-generation TKIs including osimertinib. Osimertinib is the preferred initial treatment for patients with advanced EGFR-positive NSCLC. The first-generation EGFR-TKIs, such as icotinib, are widely employed to inhibit EGFR activity in an ATP competitive and reversible manner. Nonetheless, resistance to the first-generation EGFR-TKIs develops in nearly all cancer patients [17]. Throughout time, second-generation EGFR-TKIs possessing stronger inhibitory activity against EGFR have been developed and put into practice. The covalent attachment of these TKIs to EGFR in Cys797 residues could lead to irreversible EGFR kinase inhibition. The second-generation EGFR-TKIs, including afatinib, neratinib and dacomitinib, showed superior antitumor activity to the first-generation EGFR-TKIs. However, the efficacy of these EGFR-TKIs remains limited due to frequent drug resistance (especially in response to the T790 M EGFR mutation). Osimotinib, the most successful third-generation EGFR-TKI, was developed for the treatment of locally advanced or metastatic NSCLC with the T790 M EGFR mutation or other active mutations. Osimertinib irreversibly binds to Cys797 in the EGFR kinase domain, exhibiting stronger antitumor activity than other EGFR-TKIs, which overcomes resistance induced by the T790 M mutation [17].

Genotyping NSCLC patients provides valuable information about their tumours. The prevalence of EGFR mutations is much higher in Asian people than in Caucasian or African people, and NSCLC patients with EGFR mutations are more likely to develop NSCLC-LM [18]. The most common alterations in EGFR include exon 19 deletions and the L858R point mutation in exon 21, which collectively account for 80 %–90 % of EGFR mutations [19]. The L858R mutation promotes the active conformation of the αC helix of the EGFR protein, destabilizing the inactive state [20]. TKIs block the magnesium-ATP-binding pocket of the intracellular tyrosine kinase domain. The presence of these mutations is associated with sensitivity to EGFR TKIs. The remaining 10 % of EGFR mutations are considered rare mutations, and they show variable TKI sensitivity [21], with some being sensitive (e.g., S768I, G719X, L861Q, and kinase domain duplications) [6,[10], [11], [12]], while others show comparatively poor responses to first- and second-generation EGFR TKIs (e.g., most EGFR exon 20 insertions) [[21], [22], [23]].

S768I is an uncommon mutation and accounts for 1.5 %–3 % of untreated EGFR-mutated tumours [24,25]. Patients harbouring S768I exhibit good TKI treatment responses and prognosis [26]. Multiple studies have shown that afatinib is effective for NSCLC patients with the S768I mutation [27].

T790 M mutation, as the gatekeeper EGFR mutation, is the predominant mechanism of developing resistance to TKIs, occurring in up to 50–70 % of cases [[28], [29], [30]]. T790 M alters the enzymatic activity of EGFR and the drug binding site by increasing the affinity of the EGFR protein for ATP, which inhibits tumour cell apoptosis. Osimertinib could bind to the EGFR binding site with the T790 M mutation and inhibit EGFR activity. However, resistance to osimertinib is still inevitable after long-term use. If the patient develops osimertinib resistance, there are limited treatment options.

Acquiring more than 2 mutations in the EGFR gene at the same time is defined as a compound mutation. In the present study, two cases of NSCLC-LM harboured EGFR mutations, in which one patient had an EGFR T790 M/L858R compound mutation and the other patient had an L858R/S768I compound mutation. However, few clinical data are available about the effects of these compound mutations.

Recent studies have shown that patients with L858R/T790 M compound mutations have an evaluable response to osimertinib, but not a great response, which might be because the concurrent presence of two activating mutations reverts its affinity to comparable wild-type EGFR levels. Afatinib works well for patients with compound mutations (i.e., G719X, L861Q, S768I, EGFR fusions, and kinase domain duplications) [[21], [22], [23],[30], [31], [32], [33]].

Recently, Yang et al. conducted a *post-hoc* analysis of 1023 cases and showed that afatinib demonstrated activity against major uncommon mutations and compound mutation. In this study [31], 38.6 % of patients had a compound mutation. In both TKI-naïve and EGFR TKI-pretreated patients, compound mutations were more common in the T790 M category and less common in the exon 20 insertion category. Patients with uncommon mutation and compound mutation had longer time to treatment failure. And in another retrospective study focusing on compound mutations, 125 NSCLC patients received afatinib showed longer progression-free survival than gefitinib and erlotinib, and longer overall survival than erlotinib [34].

In our current two cases with compound mutations, the patients responded well to afatinib, indicating that afatinib might be a good choice for these patients. Therefore, when patients harbour compound mutations and show resistance to first- and third-generation TKIs, afatinib should be considered an option for treatment.

In conclusion, liquid biopsy and next-generation sequencing have unique advantages for monitoring NSCLC patients. When NSCLC-LM patients with compound mutations (L858R/T790 M and L858R/S768I) show resistance to first- and third-generation TKIs, afatinib might be a good candidate for controlling their disease.

## Data availability statement

The authors confirm that the data supporting the findings of this study are available within the article [and/or] its supplementary materials. According to the Chinese legislation [*Rules for the Implementation of the Regulations on the Management of Human Genetic Resources*, promulgated on May 26th, 2023, implemented on July First 2023, URL https://www.most.gov.cn/xxgk/xinxifenlei/zc/gz/202306/t20230601_186420.html], we are not allowed to deposit or upload patients' genetic information on any oversea database, except already getting permission from the Ethics Committee of our University.

## CRediT authorship contribution statement

**Guangrui Li:** Conceptualization, Data curation, Formal analysis, Funding acquisition, Investigation, Methodology, Project administration, Resources, Validation, Writing – original draft, Writing – review & editing. **Mei Fang:** Data curation, Formal analysis, Funding acquisition, Investigation, Methodology, Resources, Software, Validation, Visualization, Writing – original draft. **Yazhu Zhou:** Data curation, Formal analysis, Investigation, Methodology, Resources, Software, Validation, Visualization. **Xiaocui Liu:** Data curation, Formal analysis, Investigation, Methodology, Validation, Visualization. **Panpan Tian:** Data curation, Formal analysis, Investigation, Methodology, Validation. **Fengjun Mei:** Conceptualization, Data curation, Formal analysis, Funding acquisition, Investigation, Methodology, Project administration, Resources, Software, Supervision, Validation, Visualization, Writing – original draft, Writing – review & editing.

## Declaration of competing interest

The authors declare that the research was conducted in the absence of any commercial or financial relationships that could be construed as a potential conflict of interest. This research is supported by 10.13039/501100001809National Natural Science Foundation of China (NSFC), Program for young scientist, No. 82201973; 10.13039/100016083Hebei Provincial Department of Human Resources and Social Security, Hebei Provincial Clinical Medical Excellent Talents Training Project, Jicai Prepayment [2021] No. 379; 10.13039/100016083Hebei Provincial Department of Human Resources and Social Security, Project for Returned Overseas Chinese Talents， No. C20210111; Hebei Provincial Department of Human Resources and Social Security, Project for Returned Overseas Chinese Talents, No. C20220109; Health Commission of Hebei Province, Hebei Provincial Research Project for Medical Science, No 20210152; Health Commission of Hebei Province, Hebei Provincial Research Project for Medical Science, No 20210079.
